# Assessing Iranians’ parental competence: Development and psychometric properties of the Children’s Sexual Behavior Questionnaire (CSBQ-IR), Iranian Version

**DOI:** 10.15171/hpp.2019.18

**Published:** 2019-05-25

**Authors:** Effat Merghati-Khoei, Fatemeh Atoof, Fatemeh Sheikhan, Sanaz Omati, Noura Aghajani, Mohsen Hosseinzadeh

**Affiliations:** ^1^Iranian National Center for Addiction Studies (INCAS), Tehran University of Medical Sciences, Tehran, Iran; ^2^Departments of Biostatistics and Epidemiology, Kashan University of Medical Sciences, Kashan, Iran; ^3^Department of Midwifery, Khalkhal Branch, Islamic Azad University, Khalkhal, Iran; ^4^Brain and Spinal Cord Injury Research Center (BASIR), Neuroscience Institute, Tehran, Iran; ^5^Islamic Azad University of Central Tehran Branch, Tehran, Iran

**Keywords:** Sexuality, Children, Iranian Parents, Questionnaire

## Abstract

**Background: ** Parents are the first line mediators in sexuality development of children. However, the majority of parents tend to have close supervision on children sexual behaviors, particularly in the conservative cultures. This article describes the development and psychometric evaluation of an instrument to measure Iranian parents’ competence in caring and nurturing their children sexually.

**Methods: ** The questionnaire was designed based on the principles in measurement, Waltz’stheory. The Iranian version of Children’s Sexual Behavior Questionnaire (CSBQ-IR) was developed and completed by 386 mothers and 101 fathers who participated in a community based sexuality education program in Tehran, capital of Iran. Reliability was assessed by Kuder-Richardson reliability coefficient and Split half. CSBQ-IR was evaluated for its construct, inclusiveness and content validity by principal component analysis.

**Results:** The Kuder-Richardson reliability coefficient and Split half reliability were found 0.425 and 0.457 that was on acceptable range. Meaning, grammar, wording and item allocation of the questionnaire were found to be appropriate with (content validity ratio [CVR]=0.99) and(content validity index [CVI]=0.8) respectively.

**Conclusion: ** CSBQ-IR provides a valid assessment of the parents’ competency or incompetency in nurturing, communication, and managing their children’s sexual behaviors.

## Introduction

Sexual behaviors are developmentally normative among children.^1,2^ The type and frequency of these behaviors vary and depend on the child’s age.^2,3^

Parents are known as the first line mediators in sexuality development of children.^4^ The majority of parents tend to have supervision on children sexual behaviors concerning its occurrence in children,^1^ particularly in non-Western societies,^5^ such as Iranian culture. Parents’ constrained views, family stressors, cultural origins and day care arrangements are the most influencing indicators of sexual behavior.^6,7^ Therefore, the parents are suggested to employ nurturing guidance about their children sexual behaviors. Some parents overreact to their child’s sexual behaviors. Children are affected by their parent’s distress toward their behaviors with sexual nature.^6^ Some of physical, psychological and sexual abuses may happen during the parenting of a child who displays sexual behavior. Some parents may be comfortable with their child’s sexual behaviors but they are not knowledgeable or able to manage those behaviors.^7^

Parents should be involved in their children’s sexuality as the first line educators.^8^ In addition to strengthening a bond between parent and child, education on sexuality would help the child to build up necessary skills and his/her highest capacity. While most parents tend to be their children’s primary sexuality educators, they sometimes experience difficulties discussing topics related to sexuality with their children.^9^ According to the studies, when parents are empowered to become their child’s primary sexuality educator, it has great impacts on increasing parent-child communication and helping children in experiencing healthy sexual life.^10-13^ It is important parents to be competent enough to detect an abnormal sexual behavior of their children.^14,15^

In some contexts parents seem not to be competent enough to nurture, communicate, care, or to implement sexuality education for children and Iranian parents are not exception.^5^ However, in the realm of sex education Iran is evidently different from most other countries or Westernized societies. For instance, some assume that little boys should not have erections so they can be protected from early sexual awakening.^5^

There are a limited number of measures to evaluate children’s sexuality. The majority of tools related to child sexuality are designed around the problematic sexual behaviors such as unwanted childhood sexual experience questionnaire^16^ childhood sexual abuse scale^17^and early sexual experiences.^18^

Brick and Koch’s questionnaire assesses the knowledge, attitudes/belief, or comfort level of professions, parents, students, or other groups of adults regarding young children’s sexual development and learning. This instrument is being useful for adults with different backgrounds in various settings which would help to further establish the psychometric properties and norms for the scales. This questionnaire contains three parts including assessing knowledge of young children’s sexual development and learning, evaluating caregivers’ attitudes and beliefs about young children’s sexual development and listing ten topics that adults typically require to talk about or deal with when interacting with young children.^19^ A sexual history and adjustment questionnaire is another tool related to children’s sexuality which was introduced by Lewis and Janda to assess parental attitudes toward sexuality, participants’ level of comfort in discussing sexuality with parents and perceptions of parental discomfort regarding sexuality. Information on current adjustment and sexual behavior was also obtained.^20^ The reliability of the sexual history and adjustment questionnaire was validated over a 15-year period. In comparison with these mentioned scales, Children’s Sexual Behavior Questionnaire (CSBQ-IR) assesses the parent’s competence in intervening and management of their children’s sexual behaviors rather than merely examining sexual behaviors of children.

In these studies emphasis was put on the assessment of parents’ sense of competence in various domains and hence a valid and reliable tool to measure this competency is needed. Aiming to contextualize Iranian parents’ competency in managing sexuality education for children, this current study was designed to develop and to test the psychometric properties of eighteen-item questionnaire to measure the parents’ knowledge, attitudes and practice toward caring and managing of their children’s sexual behaviors.

## Materials and Methods

The questionnaire was designed using the principles in measurement theory^21^ the following four steps:

*Step 1:* through an extensive review of the relevant literature, the concept of children’s sexual behavior was explored.

*Step 2:* the items of this questionnaire were generated along with a curriculum development which was planned to be implemented in a community-based sexuality education program targeting parents in Tehran. The curriculum was designed for a 6-hour’ workshop including:

a. Basic concepts about sexuality development of children extracted from stage 1

b. Common sexual behaviors in different age groups

c. Appropriate parental interventions to manage children’s sexual behaviors and educate them if needed.

The initial version of the questionnaire had 20 items. Each item was rated on a two-point response (Yes = 1; I don’t know = 0; No = 0). Closed questions evoking a response of one (Yes or No) is convenient in its own because elucidates and retrieves specific information and facts.^22^ Items 8, 9, 11, 15 and 17 were reversed questions. Higher scores indicated acceptable competency of parent in nurturing and managing children’s sexual behaviors.

*Step 3:* After preparing the items in the questionnaires, principal component analysis (PCA) was used for evaluating of validity; face validity, Content validity and construct validity.

*Step 4:* At this step, we evaluated the reliability of the questions.

The final version of questionnaire was translated and back translated by two of the authors (EMK and FS). The English version reviewed by a native English speaker for the editorial issues.

### Design and data collection

The study was carried out in three geographic zones in Tehran (North, West, East) using convenience sampling from August 2015 to May 2017. Parents voluntarily enrolled in the workshops held every Friday in the community-neighbor centers supported by the city council. Couples were eligible to attend the workshops and enter to the study if they had at least one child under age 12 or mother was pregnant for her first child. The objectives of the research were explained to them and written consent form obtained prior to the completion of questionnaire. The participants (N=487; mothers=386 [79.3%], fathers=101 [20.7%]) completed the questionnaire separately. The sample size with 50 and 1000 participants were considered sufficient and excellent in exploratory factor analysis studies.^23^

### Statistical analysis

Psychometric properties of the 18-item scale were assessed with several statistical tests.

### Reliability

The internal consistency of the questionnaire was assessed using the Kuder-Richardson reliability coefficient and split half reliability.

### Validity

At first place, 30 parents were asked to assess the questionnaire to indicate if they felt difficulty or ambiguity in responding to the questionnaire. We assessed content, inclusiveness and construct validity of the questionnaire. For the construct validity, a researcher must ascertained by a number of evidence such as content analysis, factor analysis, convergent validity (correlation coefficients) or discriminant validity (differences between differential groups or pretest-posttest intervention studies, multi-trait/multi-method studie.^24,25^

In this study, we evaluated construct validity using factor analysis and discriminant validity. Factor analysis was determined using PCA with varimax rotation to extract the factor structure of the questionnaire. In order to do discriminant validity; for conducting a contrasted group approach, study participants were divided into subgroups based on educational level and number of children. It was assumed that parents with a lower level of education and low children would attain a lower score. Differences between groups were examined using Mann-Whitney U test. R software was used in order to analyze the data.

Inclusiveness validity is defined as whether the scale appears to measure what it is supposed to measure.^21^ Content validity was examined by an expert panel consist of 15 members specialized in sexual health field for meanings, wording, item allocation and scaling of the questionnaire.^26^ To determine the validity of the instrument, we used content validity ratio (CVR) and content validity index (CVI).

## Results

### The sample

The mean age of participants, was 33.44 ± 5.27 years. The demographic characteristics of the participants are shown in Table 1. The total score for each participant was calculated by summing the individual item scores. The mean total scores in mothers and fathers were 43.3±3.9 and 43.32 ± 4.2 respectively and there was not significant difference between mean total scores of mothers and fathers (*P* value = 0.99).

### Reliability

Reliability was assessed using the internal consistency. The Kuder-Richardson reliability and spit half reliability coefficient for the questionnaire were (0.425) and (0.457) that are on the acceptable range.

### Validity

The results of content validity showed that meaning, grammar, wording and item allocation of the developed scale were found to be appropriate. For inclusive validity all participants acknowledged that total statements were easy and they had no problems in understanding the items.

*Discriminant analysis*

Also the final score was compared between subgroups of the participants’ educational level. As anticipated parents with a lower educational level scored significantly (*P *< 0.01) lower than the others 7.77 (SD 2.7) for those with under high school diploma and diploma vs 8.61 (SD 2.65) for those with up to diploma or higher. In most cases educated parents obtained their sexually related information from networks, press and media. Furthermore, parents with ≤1 children had significantly (*P *< 0.01) lower scores 7.91 (SD 2.94) for parents with <1 child, vs. 9.06 (SD 2.98) for those with 2 or more children. This suggests that parents with more than one child gained understanding of sexuality of children though lived experiences with the first and subsequent children.

*Factor analysis*

Using factor analysis with the PCA and varimax rotation, regarding Keiser criteria (eigenvalues >1) on 487 participants, a 7-component structure was found for the questionnaire items jointly accounting for 54.7% of the observed variance. But, 7-component solutions tended to produce many complex variables (an item that is in the situation of cross loading^27^ and components that had 2 or fewer variables (as a general guide, rotated factors that have less than 3 variables are not reliable and should be interpreted with caution.^28^ However, Keiser criteria may result in overestimation in the number of components extracted^27^; therefore, it is suggested to use the Scree test in conjunction with the eigenvalues to determine the number of factors to retain.^28^ So we used from this test and it indicted that a four-component solution was appropriate, and the percent of variance accounted for by a four-component solution is about 54%. The Kaiser–Meyer–Olkin measure of sampling adequacy was 0.77 and the Bartlett’s test of sphericity was found to be highly significant (χ^2^= 10048.28, *P *< 0.001, *df *= 153). These results demonstrated that the questionnaire was appropriate for factor analysis and the items could be summarized into 4 components. These components comprised the following:

Component 1 (Knowledge) including Q3, Q4, Q6, Q8, Q11, Q14

Component 2 (Educational agent) including Q5, Q12, Q13, Q16, Q18

Component 3 (belief) including Q7, Q8, Q9, Q10, Q11, Q12, Q14, Q15, Q16, Q17, Q19

Component 4 (Practice) including Q10, Q18, Q19, Q20

The loading matrix for patterns identified in the questionnaire is shown in Table 2. For the ease of interpretation, we sorted loadings by size to display the loadings in a descending order and deleted small loadings using an absolute value below 0.32 as a poor loading (2**).

## Discussion

The analysis of the CSBQ-IR dimensions revealed satisfactory psychometric properties which is a good result. All components of this tool conceptualize the competency parents need to attain. The questions put emphasis on responsibilities the literature has already pointed out^29^ including parental supervision, guidance, and addressing children educational needs such as body knowledge, rules and boundaries, respecting and protecting body privacy, sexual safety, and the means of sexuality education. The main idea for the CSBQ-IR stemmed from empirical consideration in respect to the Iranian parents’ beliefs, attitude, knowledge and practice about sexual behaviors of children.^5,7^ In the context like Iran, children sexuality facets still lack enough evidence and theoretical derivation, or culturally sensitive exploration.^30^ Brick and Koch’s questionnaire^19^ on young children’s sexual learning domains seems similar to CSBQ-IR; it may not be culturally appropriate for Iranian parents. CSBQ-IR with 18 items is easy and a brief tool to apply before educational workshops for the parents. Lewis and Janda’s sexual history and adjustment questionnaire, another tool in children’s sexuality focused on the childhood sexuality experiences such as nudity and sleeping in the parental bed^20^ while CSBQ-IR assesses the parent’s competence in intervening and management of their children’s sexual behaviors rather than examining sexual behaviors of children. In other words in contrast with the mentioned tools, target in CSBQ-IR is parents having child in 0-12 age group not children themselves.

Internal consistency assessed by Cronbach’s alpha was acceptable and indicated good reliability and stability for the CSBQ-IR. However, we do not claim that this tool is powerful enough to draw a thorough picture of the parents’ competency. Factors which may influence the validity and reliability of CSBQ-IR include nature of questions and the form of responses affected by the participants’ cognitive level. As Ziyaeemehr mentioned, CSBQ-IR questions and three points response might not challenge parents to interpret, analyze, or manipulate their own responses.^31^ According to Bloom’s taxonomy of questioning CSBQ-IR tends to measure only three levels of the parents’ cognitive processing (knowledge, comprehension, and application).^32^ A PCA with varimax rotation confirmed its factorial validity and homogeneity (Table 2). All factors showed eigenvalues above 0.3 which indicated that it did explicate enough variance to be considered. The results of the study showed that the development of a valid tool such as CSBQ-IR is necessary to assess parent’s competence in caring and managing their children’s sexual behaviors.

### Limitations

This study had some limitations. Firstly, we have not tested convergent or known-groups validity. Using a three-point response in this tool is another drawback by which we changed the design into the Liker-form employed in our currently ongoing study. In addition, the majority participated in our study had tertiary education. None-educated parents might have revealed different picture of parental competency. The result for exploratory factor analysis suggest further research with parents in a larger scale with maximum variation demographically to provide a proper measure to examine parental competence in children’s sexual behavior management.

## Conclusion

This is the first study to validate CSBQ-IR on a sample of Iranian parents. It can be a valid pre-assessment tool to screen the parents’ competency in management of their children’s sexual behaviors in order to meet the objectives of curriculum we developed to train and prepared the parents in sexuality education of their children. The recommendation for practical implications and policy making can be improvement and systematizing parental training in children sexuality education; expanding training approaches to encourage active parenting and promote parent-child communication skills as the essentials in sexually-related nurturing and caring of children.

## Ethical approval

The Ethics Committee of the Industry & University Collaboration Office at Tehran University of Medical Sciences approved the study (Ethics No. 28191-159-01-94).

## Competing interests

The authors declare that they have no competing interests.

## Funding

This research was funded by the Mental Health Department of Iranian Ministry of Health and approved by the Health Technology Development Office, Tehran University of Medical Sciences (Code: 28191-159-01-94).

## Authors’ contributions

As the primary investigator, the first author (EMK) developed the proposal and had full supervision on the whole research project as well as drafting the manuscript. The second author (FA) had full supervision on data management process and conducted the advance analysis. The third (FSH) contributed in writing the manuscript and submission follow ups. The fourth and (SO) and fifth authors (NA) had sufficient contribution in data collection and data entering phase. The last (MH) conducted further data analysis to fulfill the reviewing process.

## Acknowledgments

Our special thanks to the parents who participated in this research. We sincerely appreciate Dr. Keith Masnick’s priceless input in this manuscript. We also appreciate Dr. Razieh Maasoumi, our pos-doctorate at the time of data collection for her assistance through data entry.

**Table 1:** Demographic characteristics of the study sample (N = 487)

**Variables**	**Mother, n = 386**	**Father, n = 101**	**Total, N = 487**
Age, mean ± SD	32.75 ± 4.84	36.34 ± 5.97	33.44 ± 5.27
Educational status, No. (%)			
Under diploma and diploma	69 (17.9)	15 (14.9)	84 (17.2)
Up diploma and Bachelor	237 (61.4)	57 (56.4)	294 (60.4)
Master and PhD	80 (20.7)	29 (28.7)	109 (22.4)
Occupation, No. (%)			
Unemployed/housewife	226 (58.5)	5 (5)	231 (47.4)
Office employed	120 (31.1)	61 (60.4)	181 (37.2)
Non-office employed	40 (10.4)	35 (34.6)	75 (15.4)
Number of children, No. (%)			
≤1	250 (64.8)	63 (62.4)	313 (64.3)
2 or more	136 (35.2)	38 (37.6)	174 (35.7)

**Table 2:** Factor loading matrix for patterns identified in the questionnaire

**Items**	**Factor 1**	**Factor 2**	**Factor 3**	**Factor 4**	**Mean ± SD**
8. Is playing with genitalia instead of her/his toys normal?	-0.698		0.306		2.68 ± 0.58
6. Is watching the peer’s genitalia normal?	0.77				2.07 ± 0.84
14. Is self-stimulation in children aged 5-7 years normal?	0.369			-0.566	1.58 ± 0.66
3. Is touching and stimulating genitalia is normal for children?	0.740				2.43 ± 0.78
11. At age 2 is enjoying nudity and showing it off normal?	0.342			0.497	2.25 ± 0.80
1. Do you think children must be banned from having sexual behaviors?					
16. Are parents as the first line sexuality educator for children up to age 12?				0.468	2.81 ± 0.47
15. Do you think teacher is the first line sexuality educator for children up to age 12?				0.798	2.69 ± 0.57
2. Do children basically reveal sexual behaviors?					
5. Do you think sexuality education is necessary for children?				0.658	2.78 ± 0.53
17. Do you think children’s sexual plays or behaviors implicitly occur?			0.634		2.73 ± 0.55
13. Should a child at age 3-4 know the difference between girl and boy?				0.633	2.41 ± 0.73
9. Do you believe that children should be exposed to sexuality themes via internet or satellite?				0.667	2.58 ± 0.72
12. Does a child at age 3 normally ask questions about pregnancy, childbirth and infant?		0.584		-0.304	2.25 ± 0.75
7. Do you monitor your child's sexual behaviors?			0.671		2.75 ± 0.55
10. Do you know what to do in facing with your child’s sexual behaviors?			-0.542	-0.384	1.66 ± 0.72
18. Do you speak to your child about sexuality-related matters?				0.583	1.93 ± 0.93
4. Do you confront with your child's games like 'doctor/patient or mother and father'?	0.616				2.61 ± 0.66
20-Do you think children should be prohibited from sexual behavior?				-0.782	2.44 ± 0.67
19-Do you supervise your children’s sexual behavior?				0.607	2.69 ± 0.63
Explained Variance (%)	13.87	8.49	7.28	7.06	
Eigen value	2.5	1.53	1.31	1.27	
